# Longitudinal recordings of single units in the basal amygdala during fear conditioning and extinction

**DOI:** 10.1038/s41598-021-90530-x

**Published:** 2021-05-27

**Authors:** Junghwa Lee, Bobae An, Sukwoo Choi

**Affiliations:** 1 School of Biological Sciences, College of Natural Sciences, Seoul National University, Seoul, Republic of Korea; 2 McGovern Institute for Brain Research, Massachusetts Institute of Technology, Cambridge, MA USA

**Keywords:** Emotion, Learning and memory, Neural circuits, Neuronal physiology, Synaptic plasticity

## Abstract

The balance between activities of fear neurons and extinction neurons in the basolateral nucleus of the basal amygdala (BA_L_) has been hypothesized to encode fear states after extinction. However, it remains unclear whether these neurons are solely responsible for encoding fear states. In this study, we stably recorded single-unit activities in the BA_L_ during fear conditioning and extinction for 3 days, providing a comprehensive view on how different BA_L_ neurons respond during fear learning. We found BA_L_ neurons that showed excitatory responses to the conditioned stimulus (CS) after fear conditioning (‘conditioning-potentiated neurons’) and another population that showed excitatory responses to the CS after extinction (‘extinction-potentiated neurons’). Interestingly, we also found BA_L_ neurons that developed inhibitory responses to the CS after fear conditioning (‘conditioning-inhibited neurons’) or after extinction (‘extinction-inhibited neurons’). BA_L_ neurons that showed excitatory responses to the CS displayed various functional connectivity with each other, whereas less connectivity was observed among neurons with inhibitory responses to the CS. Intriguingly, we found correlative neuronal activities between conditioning-potentiated neurons and neurons with inhibitory responses to the CS. Our findings suggest that distinct BA_L_ neurons, which are responsive to the CS with excitation or inhibition, encode various facets of fear conditioning and extinction.

## Introduction

A neutral conditioned stimulus (CS) gains negative valence when it is paired with a noxious unconditioned stimulus (US) (fear conditioning)^1–3^. Conditioned fear can be extinguished by repeatedly presenting the CS without the US (fear extinction)^4–6^. The amygdaloid complex is implicated in these processes, and consists of several sub-nuclei, each of which has a distinct function in fear conditioning or extinction^7–17^. Among them, the basolateral nucleus of the basal amygdala (BA_L_) is thought to play a critical role in switching between low and high fear states after extinction (i.e., apparent extinction and renewal)^18–21^.

It is important to track the activity of individual neurons longitudinally throughout the whole behavioral session (i.e., conditioning and subsequent extinction) to identify the distinct populations of neurons responsible for specific stages of learning. However, there have been relatively few studies in which the activity of single neurons in the BA_L_ are monitored longitudinally during fear conditioning and extinction^21,22^. These studies focused on specific populations of BA_L_ neurons (‘fear neurons’, ’extinction neurons’ and ‘extinction-resistant neurons’), and thus, we still lack a comprehensive and unbiased picture of changes in the activity of BA_L_ neurons throughout conditioning and extinction. Fear neurons show no response to the CS before conditioning, acquire excitatory responses to CS after conditioning, and lose them after extinction. Extinction neurons show no response to the CS before or after conditioning, but acquire excitatory responses to CS after extinction. Extinction-resistant neurons show no response to the CS before conditioning, acquire excitatory responses to the CS upon conditioning, and show persistent CS-responsiveness after extinction. Since fear and extinction neurons first described in a study where single session of extinction was conducted^21^, it is unknown whether the changes observed after extinction persist when extinction is repeated. An et al. have found that extinction neurons lose their excitatory responses to the CS when extinction is repeated over multiple sessions^22^. However, it is not clear how extinction-resistant neurons change after multiple sessions of extinction. Moreover, previous studies have focused mainly on BA_L_ neurons that are excited by the CS, but not BA_L_ neurons that are inhibited by the CS. Thus, it is important to explore other types of BA_L_ neurons that are associated with conditioning and extinction.

The BA_L_ has been shown to be involved in the expression of conditioned fear after extinction^21^. Lesioning or inactivation of the BA_L_ alone before extinction does not affect the expression of conditioned fear^17–19^. Inactivation of the BA_L_ produces a significant effect on the expression of conditioned fear after extinction or upon renewal of extinguished fear^20,21^. Since the expression of conditioned fear is modulated in a context-dependent manner after extinction, it is likely that the BA_L_ is involved in this context-dependent modulation, but not in the storage of conditioned fear memory, nor in expressing this memory behaviorally, as suggested by a previous report^21^. Indeed, researchers have investigated whether the inactivation of extinction neurons or fear neurons predictably altered conditioned fear. Extinction is attenuated when infralimbic prefrontal cortex-projecting neurons, including extinction neurons, are optogenetically inactivated, and extinction is enhanced when prelimbic prefrontal cortex-projecting neurons, including fear neurons, is optogenetically inactivated^20^. These data are consistent with the proposal that the balance of activity between extinction neurons and fear neurons regulates extinction and renewal^21^.

In this study, we used fixed-microwire recordings to track longitudinal changes in the neural activity of single BA_L_ neurons during a 3-day procedure encompassing conditioning and multiple sessions of extinction. We here report that distinct sub-populations of BA_L_ neurons that encode various aspects of fear conditioning and extinction and the functional connectivity among distinct BA_L_ neurons.

## Results

### Behavioral results during fear conditioning and multiple sessions of extinction

**Figure 1:**
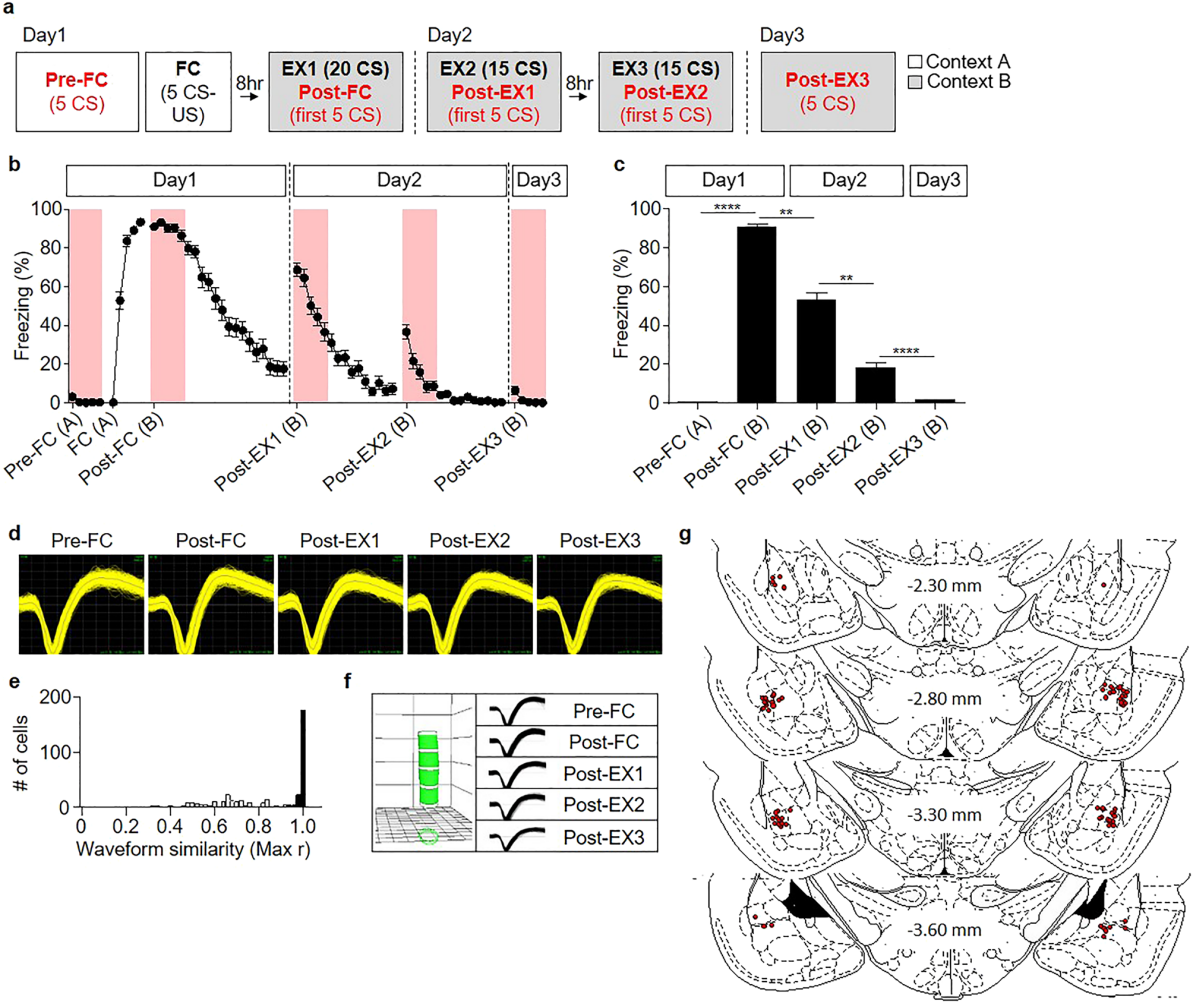
Long-term single-unit recordings in the BA_L_ throughout fear conditioning (FC) and extinction (EX). (**a**) The behavioral procedure used in the electrophysiological experiment. Pre-FC and FC sessions were conducted in context A (white) and EX1-3 and Post-EX3 were conducted in context B (gray). (**b**) Averaged learning curves of the behavioral sessions. Conditioned freezing to each CS was measured and averaged among rats throughout the behavioral training. The context where each behavioral session was taken is depicted as A for context A and B for context B. (**c**) Averaged freezing responses during the first five conditioned stimulus presentations in each session. The error bars indicate the standard error of the mean. **p < 0.005; ****p < 0.00001. (**d**) Representative waveforms of a neuron recorded from a single electrode throughout the behavioral training period. The waveforms were visualized using Offline Sorter, Version 3.3.5 (Plexon, Dallas, TX, www.plexon.com). (**e**) Quantitative evaluation of waveform similarity from the units recorded across all behavioral sessions. Randomly selected waveforms were used as controls (white bars). (**f**) Verification of the long-term stable single-unit recordings using principal component space cylinders. A straight cylinder suggests that the same set of single units has been recorded in different behavioral sessions. The space cylinders were created using WaveTracker, Version 1.15 (Plexon, Dallas, TX, USA, www.plexon.com). (**g**) Histological verification of the electrode placement in all experiments. The atlas was derived from open atlas Brain maps^44^.

A total of 41 rats underwent fear conditioning and multiple sessions of extinction, as described previously (^22,23^ see also “Materials and methods” section) (Fig. 1a) and freezing to each CS was measured to estimate their fear levels. The CS was a series of 27 pure-tone pips (200 ms duration repeated at 0.9 Hz). The behavioral and neural responses were averaged over the five CSs before conditioning (Pre-FC), the first five CSs in each extinction session (Post-FC, Post-EX1, and Post-EX2), and the five CSs in the test session (Post-EX3).

The rats, which had been handled and habituated, displayed no significant freezing before conditioning (Pre-FC). Eight hours after the initial fear conditioning, the rats showed strong freezing when they were exposed to the CS in a different context (Post-FC). The conditioned responses decreased progressively over three sessions of extinction (EX1 through EX3), and eventually returned close to baseline levels (Post-EX3) (Fig. 1b, c).

### CS-evoked single unit activities in the BA_L_

**Figure 2:**
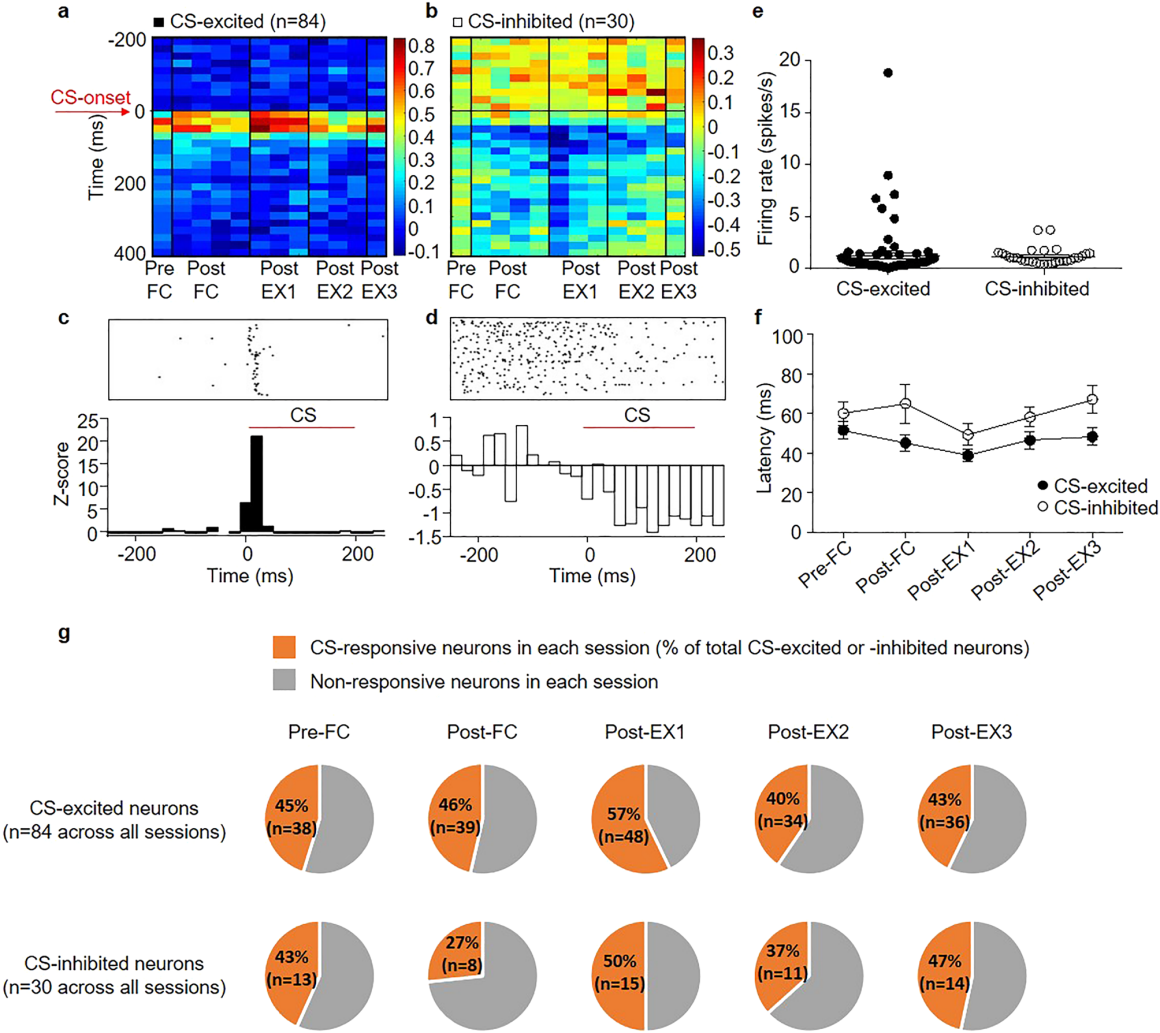
CS-evoked activities of BA_L_ neurons over the entire behavioral training. (**a**) Heatmap showing the averaged z-scores of all the CS-excited neurons (n = 84). (**b**) Heatmap showing the averaged z-scores of all the CS-inhibited neurons (n = 30). Each column in (**a)** and (**b)** represents the averaged z-scores of five consecutive CS presentations. Each row in (**a)** and (**b**) represents 20 ms. The heatmaps were created using MATLAB R2018b (Mathwork, Inc., Natick, MA, USA, www.mathworks.com). (**c**) Representative raster plot and peri-event time histogram (PETH) of a CS-excited neuron. (**d**) Representative raster plot and PETH of a CS-inhibited neuron. The raster plots were created using NeuroExplorer, Version 4.135 (Nex Technologies, Colorado Springs, CO, USA, www.neuroexplorer.com). (**e**) Spontaneous firing rates of CS-excited and CS-inhibited neurons. (**f**) Latency changes in CS-excited and CS-inhibited neurons over the entire behavioral protocol. (**g**) Pie charts showing the percentage of CS-responsive neurons among CS-excited neurons (top, n = 84 across all sessions) or among CS-inhibited neurons (bottom, n = 30 across all sessions) in a given session.

A total of 204 high signal-to-noise units in the BA_L_ were stably recorded throughout the behavioral training, which were verified using principal component and correlation analyses (Fig. 1d–f), and was included in the data analysis. Histological analysis confirmed that these units had been recorded via electrodes with tips located in the BA_L_ (Fig. 1g). 56% of recorded neurons (114 neurons) responded to the CS significantly in at least one of the training sessions (The Wilcoxon rank-sum test, *p* < 0.05). Among them, one group of BA_L_ neurons exhibited excitatory responses to the CS as compared with their baseline firing rates during a period preceding the presentation of the CS (84 neurons, 74% of CS-responsive neurons; Fig. 2a, c), whereas the other group exhibited inhibitory responses to the CS as compared with their baseline firing rates (30 neurons, 26% of CS-responsive neurons; Fig. 2b, d). The average spontaneous firing rate was 1.178 ± 0.1336 Hz for CS-excited neurons and 1.252 ± 0.1622 Hz for CS-inhibited neurons (Fig. 2e). The average onset latency, which was calculated as the interval from the CS-onset to the appearance of the first significant response, was 43.87 ± 2.280 ms for CS-excited neurons and 58.00 ± 3.566 ms for CS-inhibited neurons (*p* = 0.0276 for Post-EX3, Mann–Whitney test) (Fig. 2f). The low spontaneous firing rates with short latencies to auditory CS were consistent with the findings of previous studies^21,24–27^. In addition, the proportion of CS-responsive neurons (CS-excited or CS-inhibited neurons) varied between sessions (Fig. 2g).

**Figure 3:**
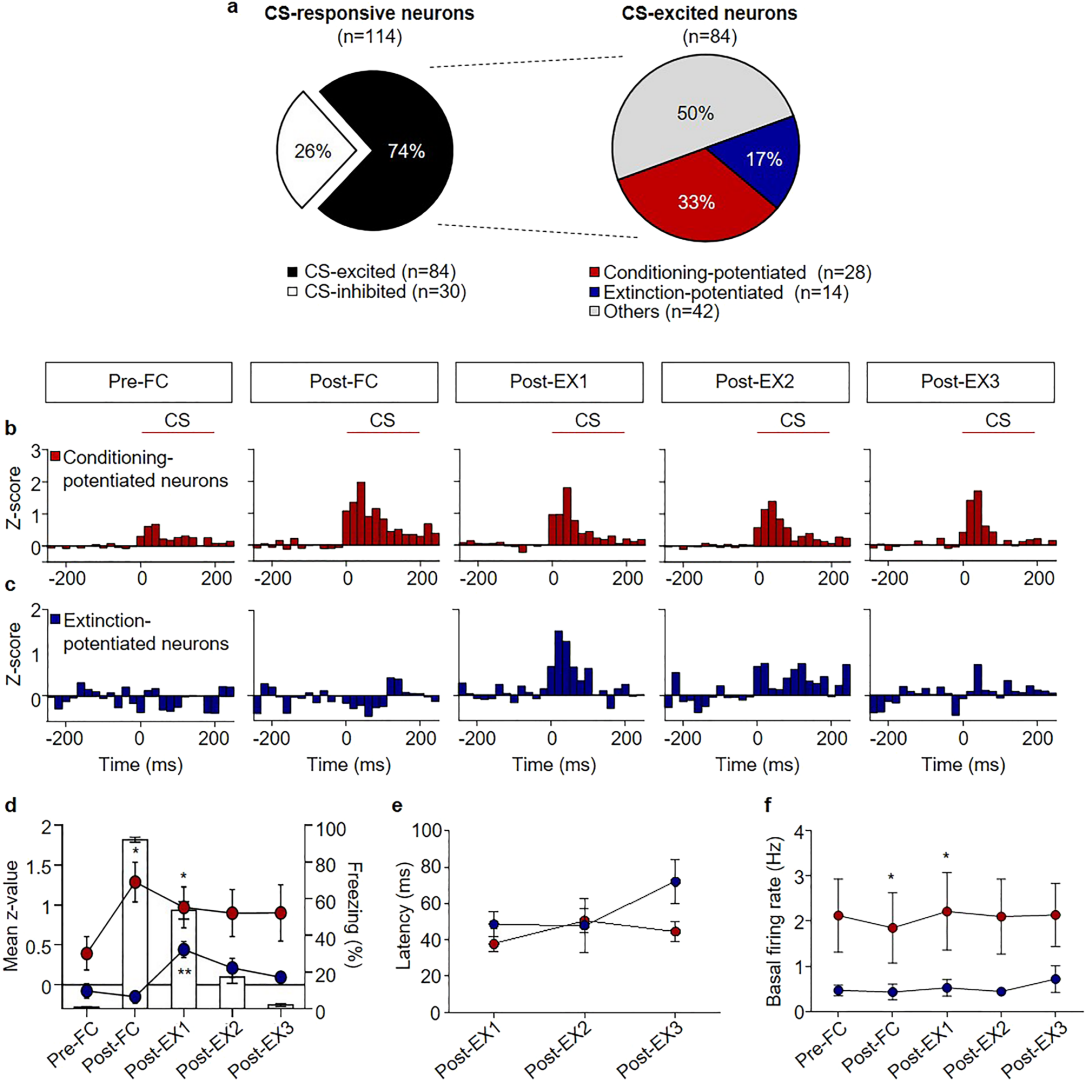
Conditioning-potentiated and extinction-potentiated neurons display potentiated responses to the CS after conditioning and extinction, respectively. (**a**) Pie chart showing the percentage of CS-excited neurons among the CS-responsive neurons (left) and the percentage of conditioning-potentiated and extinction-potentiated neurons among the CS-excited neurons (right). (**b**) Z-score PETH of conditioning-potentiated neurons (n = 28, 33% of CS-excited neurons). (**c**) Z-score PETH of extinction-potentiated neurons (n = 14, 17% of CS-excited neurons). (**d**) Averaged time courses of freezing responses and neuronal activity (z-scores) of conditioning-potentiated (red circle) and extinction-potentiated neurons (blue circle). Each bar represents the averaged freezing during five consecutive CS presentations, and each point represents the averaged z-scores during five consecutive CS presentations. (**e**) Comparison of the onset response across all behavioral sessions. (**f**) Comparison of the basal firing rates of the conditioning-potentiated and extinction-potentiated neurons across all behavioral sessions. *p < 0.05; **p < 0.005.

**Figure 4:**
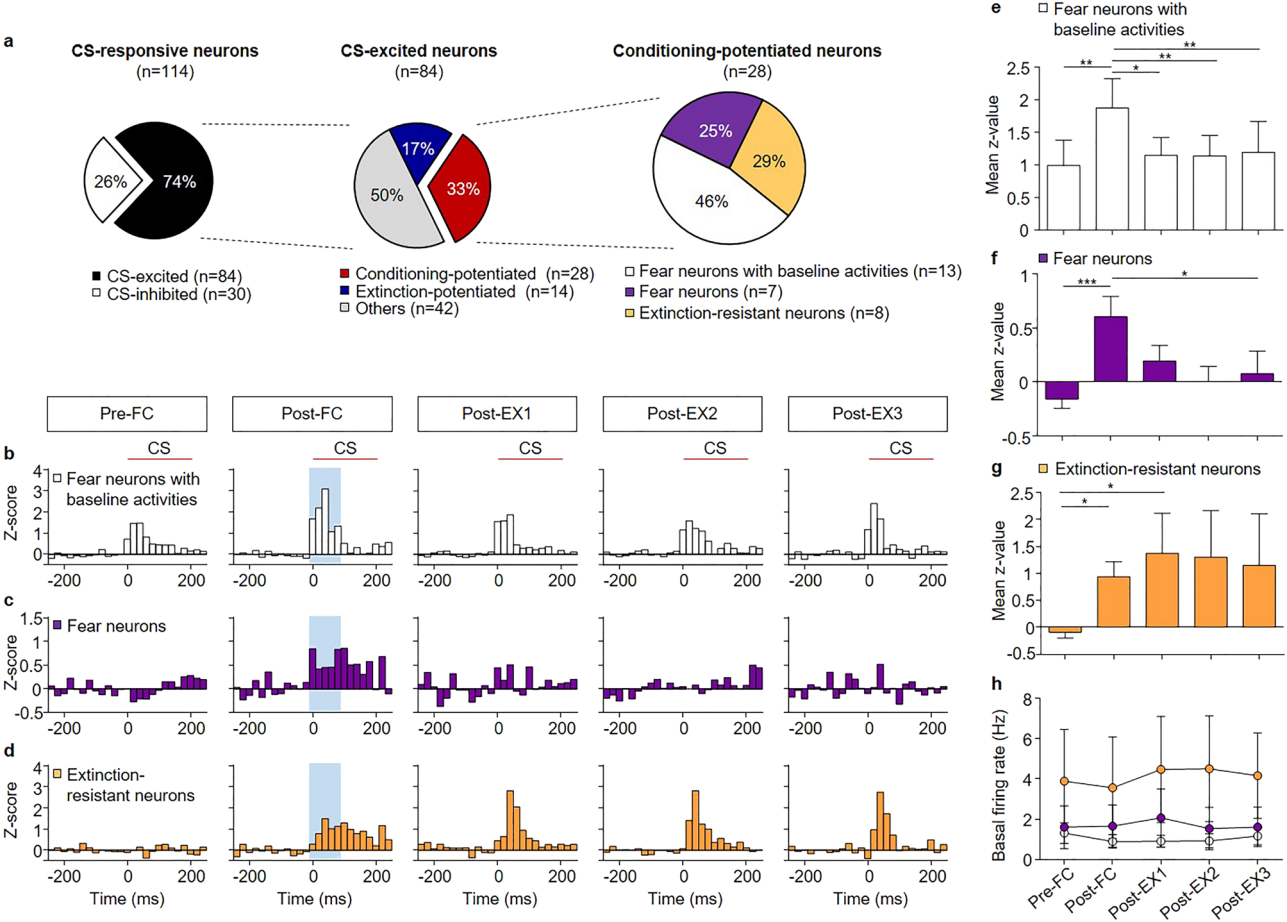
Distinct sub-populations of conditioning-potentiated neurons encode different facets of fear conditioning. (**a**) Pie chart showing the percentage of CS-excited neurons among the CS-responsive neurons (left), the percentage of conditioning-potentiated neurons among the CS-excited neurons (middle), and the percentage of three types of conditioning-potentiated neurons (right). (**b**) Z-score PETH of fear neurons with baseline activities (n = 13, 46% of conditioning-potentiated neurons). (**c**) Z-score PETH of fear neurons (n = 7, 25% of conditioning-potentiated neurons). (**d**) Z-score PETH of extinction-resistant neurons (n = 8, 29% of conditioning-potentiated neurons). (**e–g**) The mean z-score comparisons of (**e**) fear neurons with baseline activities, (**f**) fear neurons, (**g**) extinction-resistant neurons. (**h**) Comparison of basal firing rates of fear neurons with basal activities (white circle), fear neurons (purple circle) and extinction-resistant neurons (orange circle) across all behavioral sessions. *p < 0.05; **p < 0.01; ***p < 0.0005.

**Figure 5:**
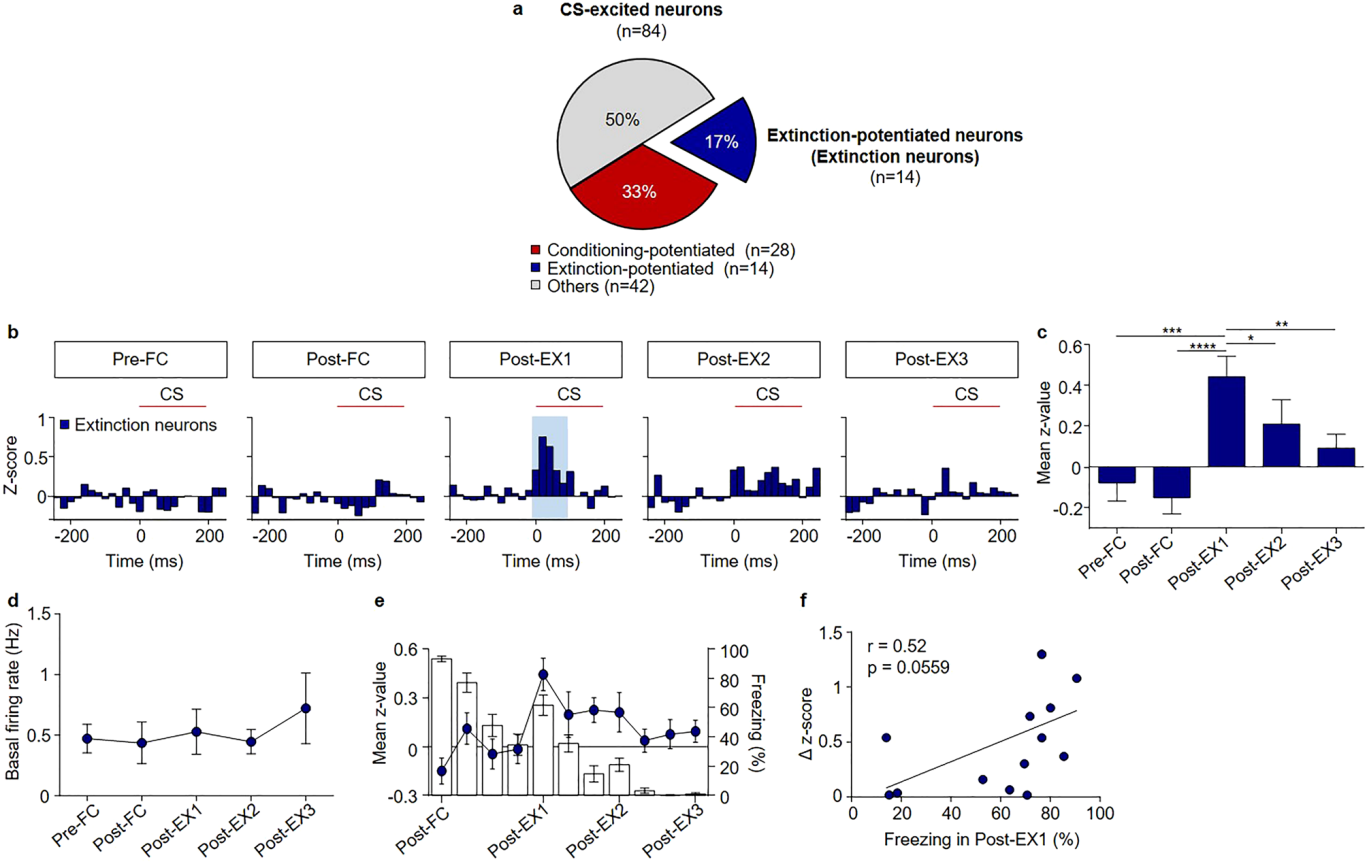
Extinction neurons encode the early phase of fear extinction. (**a**) Pie chart showing the percentage of extinction neurons among the CS-excited neurons. (**b**) Z-score PETH of extinction neurons (n = 14, 17% of CS-excited neurons). (**c**) Mean z-score comparisons of extinction neurons. (**d**) Comparison of the basal firing rates of extinction neurons across all behavioral sessions. (**e**) Averaged time courses of freezing responses and neuronal activity (z-scores) of extinction neurons. (**f**) Correlation analysis between the neural responses and freezing behavior in Post-EX1. A positive trend was observed (r = 0.52, *p* = 0.0559). *p < 0.05; **p < 0.005; ***p < 0.0005; ****p < 0.0001.

Previous reports have demonstrated that the BA_L_ plays a critical role in conditioning and extinction^16–21^. Thus, we first sought BA_L_ neurons which are displaying significant and increased excitatory responses to the CS either after conditioning (Post-FC) or after the first session of extinction (Post-EX1) when compared with the preceding session(s), and these two populations of BA_L_ neurons were defined as conditioning-potentiated and extinction-potentiated neurons, respectively (Figs. 3, 4, 5).

### CS-excited neurons

We found two distinct populations of CS-excited neurons: conditioning-potentiated and extinction-potentiated neurons (Fig. 3a). Conditioning-potentiated neurons (n = 28, from 20 rats, 33% of CS-excited neurons, Fig. 3a,b) showed larger responses to the CS in Post-FC than in Pre-FC (*χ*^2^ = 31.91, *p* < 0.0001, *p* < 0.05 for Post-FC vs. Pre-FC, Friedman test followed by Dunn’s test; Fig. 3d), and tended to remain potentiated in subsequent extinction and test sessions, although this difference was not statistically significant after multiple extinction sessions (*p* > 0.05 for Post-EX2 or Post-EX3 vs. Pre-FC, *p* = 0.0041 for Post-EX1 vs. Pre-FC).

Extinction-potentiated neurons (n = 14, from 12 rats, 17% of CS-excited neurons, Fig. 3a, c) showed larger responses to the CS in Post-EX1 than in the two preceding sessions (*χ*^2^ = 24.00, *p* < 0.0001, *p* < 0.05 for Pre-FC, Post-FC vs. Post-EX1, Friedman test followed by Dunn’s test; Fig. 3d), and the responses decreased in subsequent extinction and test sessions (*p* > 0.05 for Post-EX2, or Post-EX3 vs. Pre-FC).

On average, the latency and spontaneous firing rate of the conditioning-potentiated or extinction-potentiated neurons did not change significantly across sessions (Fig. 3e, f). The averaged latency and spontaneous firing rate of the conditioning-potentiated neurons were 44.60 ± 2.351 ms and 2.086 ± 0.3505 Hz, respectively, and those of the extinction-potentiated neurons were 53.33 ± 5.732 ms and 0.5206 ± 0.08195 Hz, respectively. The spontaneous firing rates of the conditioning-potentiated neurons were higher than the extinction-potentiated neurons after fear conditioning and the first extinction session (*p* < 0.05 for Post-FC and Post-EX1, Mann–Whitney test; Fig. 3f).

Conditioning-potentiated neurons were divided into three sub-populations based on their CS-responses before and after fear conditioning—‘fear neurons with baseline activity’, ‘fear neurons’, and ‘extinction-resistant neurons’ (Fig. 4a). Fear neurons with baseline activities, which showed CS-evoked excitation before conditioning (n = 13, from 11 rats, 46% of CS-excited neurons, Fig. 4a,b), showed larger responses to the CS in Post-FC than Pre-FC (*χ*^2^ = 12.43, *p* = 0.0144, *p* < 0.05 for Post-FC vs. Pre-FC, Friedman test followed by Dunn’s test; Fig. 4e). The larger responses in Post-FC returned to baseline activities in Pre-FC after the first session of extinction (*p* > 0.05 for Post-EX1, Post-EX2, or Post-EX3 vs. Pre-FC). Fear neurons (n = 7, from 6 rats, 25% of CS-excited neurons, Fig. 4a,c) showed no significant responses before conditioning, but developed significant responses to the CS in Post-FC (*χ*^2^ = 13.37, *p* = 0.0096, *p* < 0.05 for Post-FC vs. Pre-FC, Friedman test followed by Dunn’s test; Fig. 4f), consistent with the findings of previous studies^21,22^. The CS-evoked activities disappeared after the first session of extinction (*p* = 0.0426 for Pre-FC vs. Post-EX1, *p* > 0.05 for Post-EX2 or Post-EX3 vs. Pre-FC). Extinction-resistant neurons (n = 8, from 7 rats, 29% of CS-excited neurons, Fig. 4a, d), which showed no significant responses in Pre-FC, showed significant responses to the CS in Post-FC (*χ*^2^ = 9.200, *p* = 0.0563, *p* < 0.05 for Post-FC vs. Pre-FC, Friedman test followed by Dunn’s test; Fig. 4g), consistent with previous studies^21,22^. The CS-evoked activities remained potentiated after extinction (*p* < 0.05 for Post-EX1 vs. Pre-FC). The average spontaneous firing rate was 1.044 ± 0.1733 Hz, 1.699 ± 0.4752 Hz, and 4.118 ± 1.061 Hz for fear neurons with baseline activities, fear neurons, and extinction-resistant neurons, respectively (Fig. 4h).

Extinction-potentiated neurons (extinction neurons; n = 14, from 12 rats, 78% of CS-excited neurons, Fig. 5a,b), which showed no significant activities in Pre-FC and Post-FC, showed significant responses to the CS in Post-EX1 (*χ*^2^ = 24.00, *p* < 0.0001, *p* < 0.05 for Pre-FC or Post-FC vs. Post-EX1, Friedman test followed by Dunn’s test; Fig. 5c), consistent with previous studies^21,22^. The CS-evoked activities disappeared after the second session of extinction (*p* > 0.05 for Post-EX2 or Post-EX3 vs. Pre-FC). The average spontaneous firing rate of extinction neurons was 0.5206 ± 0.08195 Hz (Fig. 5d).

Next, we compared the averaged time courses of CS-evoked activity of conditioning-potentiated and extinction-potentiated neurons with freezing in extinction (Fig. 5e and Supplementary Fig. S1). The averaged time courses of CS-evoked activity in fear neurons and fear neurons with baseline activities were similar to those of freezing during the first extinction (Supplementary Fig. S1a, b). However, CS-evoked activity persisted during extinction in the case of extinction-resistant neurons (Supplementary Fig. S1c). These results are consistent with a previous suggestion that fear neurons are responsible for the immediate expression of conditioned fear^21^, whereas extinction-resistant neurons represent the engram of the persistent fear memory during extinction^28^. Furthermore, extinction neurons showed enhanced CS-evoked activity only in Post-EX1 (Fig. 5e), consistent with a previous study^21,22^. This indicates that extinction neurons may play a critical role only in the early phase of fear extinction.

In addition, we compared the CS-evoked activity of individual extinction neurons in Post-EX1 or the CS-responses of individual conditioning-potentiated in Post-FC with freezing in Post-EX1 to determine whether changes in BA_L_ neuronal activities are correlated with behavioral changes after extinction (Fig. 5f and Supplementary Fig. S2). The change in the activity of individual fear neurons showed a positive trend with freezing in Post-EX1 (Supplementary Fig. S2b; r = 0.65, *p* = 0.1161, Pearson’s correlation). Interestingly, changes in the activity of individual fear neurons with baseline activities showed negative, but non-significant correlation with freezing (Supplementary Fig. S2a; r = − 0.32, *p* = 0.2887, Pearson’s correlation). The changes in the activity of individual extinction neurons also showed a positive trend with freezing (Fig. 5f; r = 0.52, *p* = 0.0559, Pearson’s correlation).

### CS-inhibited neurons

**Figure 6:**
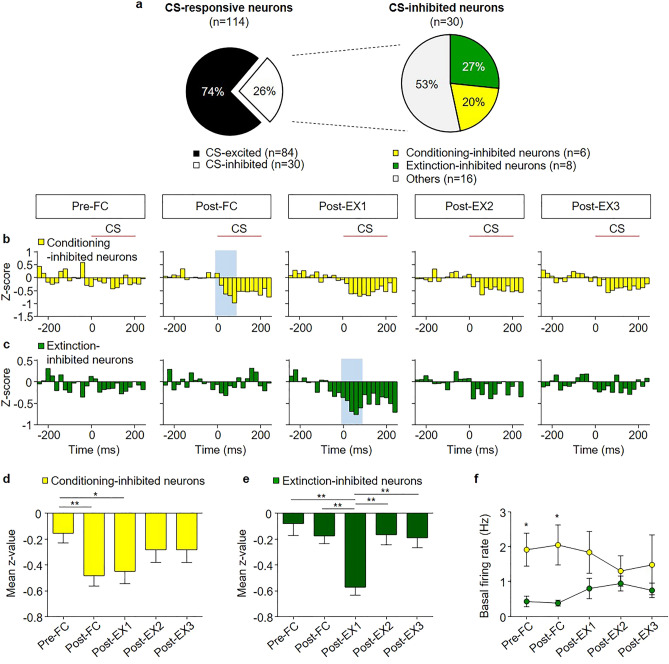
Distinct sub-populations of CS-inhibited neurons encode conditioning and extinction. (**a**) Pie chart showing the percentage of CS-inhibited neurons among the CS-responsive neurons (left) and the percentage of conditioning-inhibited and extinction-inhibited neurons among the CS-inhibited neurons (right). (**b**) Z-score PETH of conditioning-inhibited neurons (n = 6, 20% of CS-inhibited neurons). (**c**) Z-score PETH of extinction-inhibited neurons (n = 8, 27% of CS-inhibited neurons). (**d**) Mean z-score comparisons of the conditioning-inhibited neurons. (**e**) The mean z-score comparisons of extinction-inhibited neurons. (**f**) Comparison of basal firing rates of conditioning-inhibited (yellow circle) and extinction-inhibited neurons (green circle) across all behavioral sessions. *p < 0.05; **p < 0.01.

We found two distinct populations of CS-inhibited neurons: conditioning-inhibited and extinction-inhibited neurons (Fig. 6a). The conditioning-inhibited neurons, which showed no significant inhibitory responses in Pre-FC (n = 6, from 6 rats, 20% of CS-inhibited neurons, Fig. 6a, b) showed significant inhibitory responses to the CS in Post-FC (*χ*^2^ = 10.80, *p* = 0.0289, *p* < 0.05 for Post-FC vs. Pre-FC, Friedman test followed by Dunn’s test; Fig. 6d). The CS-evoked inhibition disappeared after the second session of extinction (*p* > 0.05 for Post-EX2 or Post-EX3 vs. Pre-FC, *p* = 0.0106 for Pre-FC vs. Post-EX1). This population of BA_L_ neurons is similar to the fear neurons found among CS-excited neurons.

Extinction-inhibited neurons (n = 8, from 7 rats, 27% of CS-inhibited neurons; Fig. 6a, c), which showed no significant inhibitory activity in Pre-FC and Post-FC, showed significant inhibitory responses to the CS in Post-EX1 (*χ*^2^ = 14.40, *p* = 0.0061, *p* < 0.05 for Pre-FC and Post-FC vs. Post-EX1; Friedman test followed by Dunn’s test; Fig. 6e). The CS-evoked inhibitory activities disappeared after the second session of extinction (*p* > 0.05 for Post-EX2 or Post-EX3 vs. Pre-FC). This sub-population of BA_L_ neurons is similar to the ‘extinction neurons’ found among CS-excited neurons. The average spontaneous firing rate was 1.717 ± 0.2572 Hz for conditioning-inhibited neurons and 0.6630 ± 0.09192 Hz for extinction-inhibited neurons. The spontaneous firing rates of conditioning-inhibited neurons were higher than extinction-inhibited neurons before and after fear conditioning (*p* < 0.05 for Pre-FC and Post-FC, Mann–Whitney test; Fig. 6f). These findings suggest that BA_L_ neurons, which show inhibitory responses to the CS, also modify their responses to the CS during fear conditioning and extinction, similar to the CS-excited neurons.

Subsequently, we compared the averaged time courses of the CS-evoked activity of the conditioning-inhibited and extinction-inhibited neurons with freezing during the extinction of conditioned fear (Supplementary Fig. S3a, b). The averaged time course of the CS-evoked activity of conditioning-inhibited neurons was similar to that of freezing during extinction (Supplementary Fig. S3a). In contrast, extinction-inhibited neurons showed enhanced CS-evoked activity only in Post-EX1 (Supplementary Fig. S3b). CS-responses of conditioning-inhibited neurons and extinction-inhibited neurons changed similarly to those of fear neurons and extinction neurons, respectively, throughout the behavioral training.

Additionally, we compared the CS-evoked activity of individual neurons in Post-EX1 with freezing in Post-EX1 to determine whether the magnitude of the activity changes of the individual BA_L_ neurons correlate with behavioral changes after extinction (Supplementary Fig. S3c, d). The activity changes of conditioning-inhibited neurons showed a positive trend with freezing in the recorded rats (Supplementary Fig. S3c; r = 0.76, *p* = 0.0746, Pearson’s correlation), whereas those of extinction-inhibited neurons showed a negative trend with freezing (Supplementary Fig. S3d; r = -0.40, *p* = 0.3326, Pearson’s correlation). Together, the responses of BA_L_ neurons which are inhibited by the CS are dynamically modified throughout fear conditioning and extinction, suggesting CS-inhibited neurons are also actively involved in fear learning and extinction.

### Functional connectivity among CS-responsive neurons in the BA_L_

**Figure 7:**
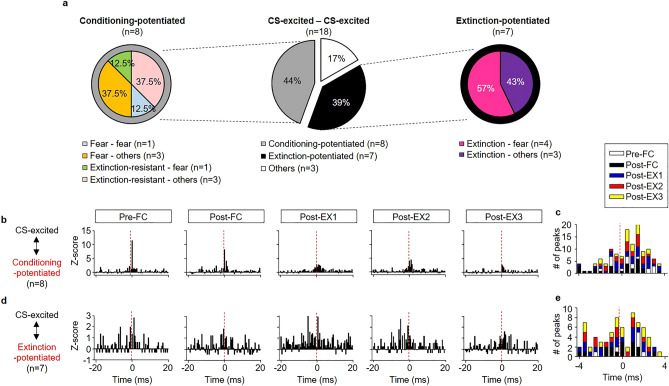
Functional connectivity between CS-excited neurons in the BA_L_. (**a**) Types of functional connectivity between CS-excited neurons (middle), between conditioning-potentiated neurons and CS-excited neurons (left) and between extinction-potentiated neurons and CS-excited neurons (right). (**b, d**) Z-score cross-correlograms between conditioning-potentiated neurons and CS-excited neurons (**b**; n = 8, 44% of CS-excited neuronal pairs, conditioning-potentiated neurons as references and CS-excited neurons as targets) and between extinction-potentiated neurons and CS-excited neurons (**d**; n = 7, 39% of CS-excited pairs, extinction-potentiated neurons as references). (**c**) The distribution of the time of significant peaks in cross-correlograms for the pairs between CS-excited neurons and conditioning-potentiated neurons. Connectivity was considered as significant if there is any peak > 99% confidence index within 4 ms in the cross-correlogram and the significant peaks were counted. Positive peaks in the cross-correlograms mean firings of a reference neuron preceded a target neuron. Conditioning-potentiated neurons tend to fire before than CS-excited neurons in the pairs. (**e**) The distribution of significant peaks in cross-correlograms for the pairs between CS-excited neurons and extinction-potentiated neurons shows synchronous firings.

**Figure 8:**
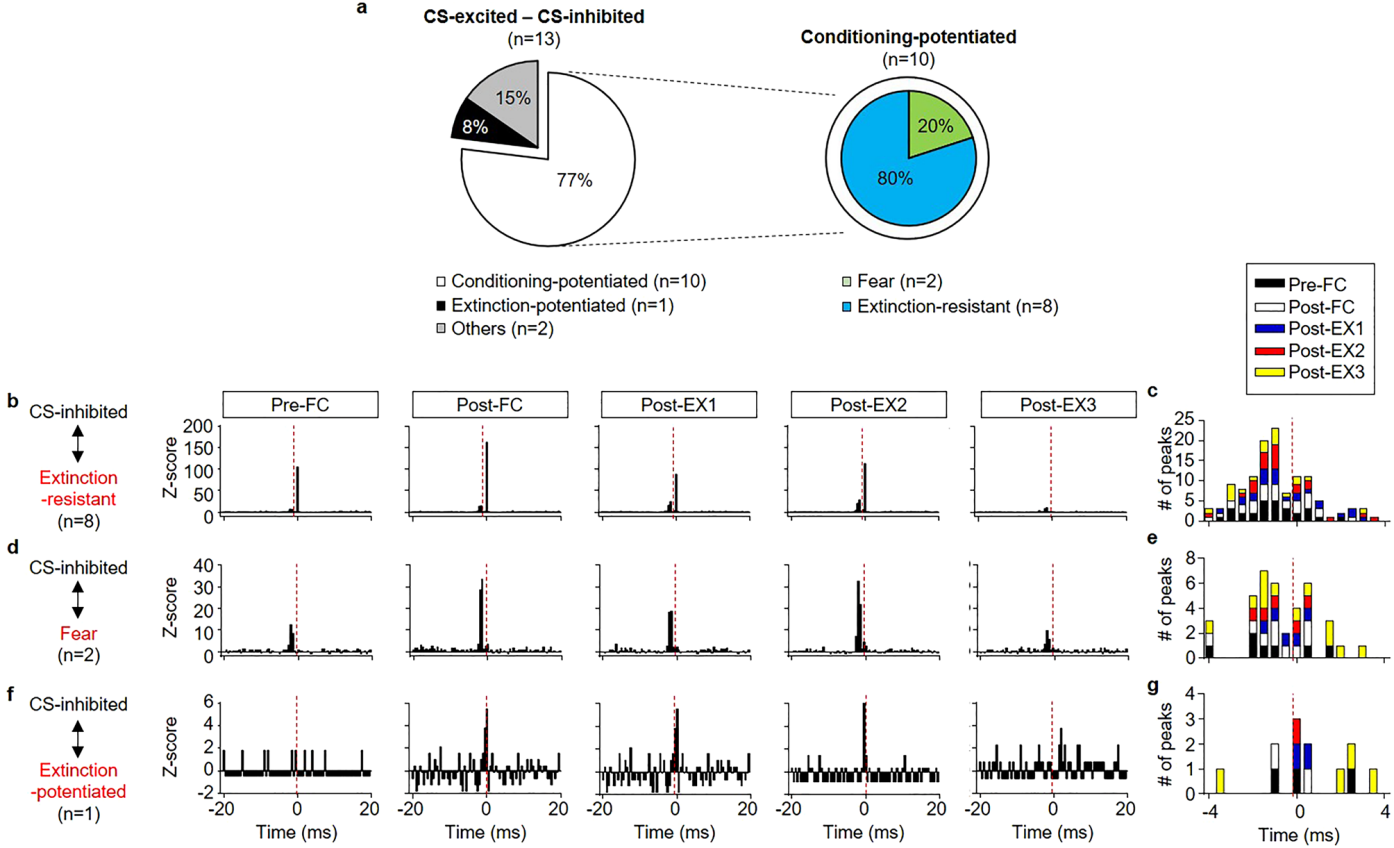
Functional connectivity between CS-excited neurons and CS-inhibited neurons in the BA_L_. (**a**) Types of functional connectivity between CS-excited neurons and CS-inhibited neurons (left), between conditioning-potentiated neurons and CS-inhibited neurons (right). (**b, d, f**) Z-score cross-correlograms between CS-inhibited neurons and extinction-resistant neurons (**b**; n = 8, 61% of CS-excited and CS-inhibited neuronal pairs, extinction-resistant neurons as references and CS-inhibited neurons as targets), between CS-inhibited neurons and fear neurons (**d**; n = 2, 16%, fear neurons as references), and between a CS-inhibited and an extinction-potentiated neuron (**f**; n = 1, 8%, extinction-potentiated neuron as a reference). (**c, e, g**) The distribution of the time of significant peaks in cross-correlograms for the pairs between CS-inhibited neurons and (**c**) extinction-resistant neurons, (**e**) fear neurons, and (**g**) extinction-potentiated neurons. CS-inhibited neurons tend to fire before than extinction-resistant neurons and fear neurons in the pairs.

We further examined whether there is any functional connectivity among various types of CS-responsive neurons in the BA_L_ and whether the connectivity changes during fear conditioning and subsequent extinction. Cross-correlation analysis between spike trains of neuronal pairs, which were simultaneously recorded throughout the behavioral training, was performed. Peaks within 4 ms in cross-correlograms were examined, as indicators of direct connectivity or synchronous firings by common inputs and any peak over 99% confidence limits within 4 ms in the cross-correlograms was considered to be significant^29–31^. The direction of the correlative firings was determined based on the time of the significant peaks in the cross-correlograms. Significant positive peaks in the cross-correlograms were considered as the activities of the reference neurons preceding those of the target neurons. A total of 112 simultaneously recorded CS-responsive neurons in 37 rats were observed, resulting in 176 possible connections. We found 18 pairs of CS-excited neurons in 9 rats that showed significant correlative firings in any of the behavioral training sessions (Fig. 7a). The functional connectivity between CS-excited neurons was various; 44% of the pairs involved conditioning-potentiated neurons (n = 8 pairs, Fig. 7b) and conditioning-potentiated neurons are likely fire before CS-excited neurons (Fig. 7c). 39% of the pairs involved extinction-potentiated neurons (n = 7 pairs, Fig. 7d) and the directions of correlated firings varied throughout the behavioral training (Fig. 7e), suggesting synchronous firings by common inputs. We also found that a few pairs of CS-excited neurons which displayed fear learning-related behavior showed correlated firings with each other (Supplementary Fig. S4a–c). Three pairs between fear neurons and extinction neurons showed synchronous firings throughout the behavioral training (Supplementary Fig. S4a, g). Two fear neurons showed synchronous firings only in Pre-FC and Post-FC (Supplementary Fig. S4b, h). One pair between a fear neuron and an extinction-resistant neuron developed functional connectivity following extinction (Supplementary Fig. S4c, i). In contrast, correlated firings between CS-inhibited neurons were rare, and we found only three pairs of CS-inhibited neurons with significant correlation in 2 rats (Supplementary Fig. S4d–f, j–l). We also examined correlated firings between CS-excited and CS-inhibited neurons and found 13 pairs in 7 rats (Fig. 8a). Intriguingly, majority of the pairs involved conditioning-potentiated neurons (77%, n = 10 pairs); 8 pairs of them involved extinction-resistant neurons (Fig. 8b) and the other involved fear neurons (n = 2 pairs, Fig. 8d). The firings of CS-inhibited neurons tend to precede the firings of conditioning-potentiated neurons, both fear neurons and extinction-resistant neurons (Fig. 8c, e). These results suggest that CS-inhibited neurons may play a crucial role in fear learning and extinction by modulating the activities of conditioning-potentiated neurons. We found only one pair involving extinction-potentiated neuron, which developed functional connectivity after fear conditioning (Fig. 8f). We also found a few pairs involved CS-inhibited neurons that displayed fear learning-related behavior (Supplementary Fig. S5a–c). One pair between a conditioning-inhibited neuron and an extinction-resistant neuron displayed increased connectivity after fear learning (Supplementary Fig. S5a, d). One pair between a conditioning-inhibited neuron and a fear neuron (Supplementary Fig. S5b, e) and three pairs between extinction-inhibited neurons and extinction-resistant neurons (Supplementary Fig. S5c, f) showed synchronous firings throughout the behavioral training. Together, these results suggest distinct BA_L_ neurons which encode various facets of fear learning and extinction show functional connectivity which may modify fear memory representation. CS-inhibited neurons may play a crucial role in modulating fear memory after extinction by modifying the activities of extinction-resistant neurons.

## Discussion

In this study, we found two sub-populations of BA_L_ neurons that exhibited either CS-evoked excitation or inhibition in fear conditioning and subsequent extinction (Supplementary Fig. S7a). We found two BA_L_ neuronal populations that were previously reported^21,22^, one that developed CS-evoked excitation following fear conditioning and lost CS-responses after extinction (fear neurons) and the other that developed CS-responses after extinction (extinction neurons). We also found new neuronal sub-populations in the BA_L_; one that showed CS-evoked excitation even before conditioning (fear neurons with baseline activities) and another population that developed CS-evoked excitation after fear conditioning and retained the potentiated responses even after extinction (extinction-resistant neurons). Furthermore, we also found BA_L_ neurons that exhibited CS-evoked inhibition; one population developed CS-responses following conditioning (conditioning-inhibited neurons) and the other population exhibited CS-evoked inhibition following extinction (extinction-inhibited neurons), and both returned to the pre-training level after multiple extinction sessions. The activity of conditioning-inhibited neurons was positively correlated with the fear response after the first session of extinction, whereas that of extinction-inhibited neurons was negatively correlated with the fear response after the first session of extinction. Intriguingly, we found correlated firings between CS-inhibited neurons and conditioning-potentiated neurons, especially extinction-resistant neurons (Supplementary Fig. S7b). These findings suggest that CS-inhibited neurons also play an important role in regulating fear states after extinction. Consequently, our findings indicate that the regulation of fear states via BA_L_ neurons may be more complex than previously thought.

There have been only relatively few studies^21,22^ in which single units in the BA_L_ are recorded longitudinally throughout the entire session of conditioning and subsequent extinction. Consistent with their findings, we have also found three populations of BA_L_ neurons (fear, extinction, and extinction-resistant neurons of CS-excited neurons) in the present study. In the previous studies, Herry et al. did not consider CS-inhibited neurons for further analysis, and An et al. focused on fear and extinction neurons. Therefore, this is the first study in which the activities of CS-inhibited neurons have been longitudinally monitored during conditioning and subsequent extinction. Intriguingly, the two sub-populations of CS-inhibited neurons in the BA_L_ resemble fear and extinction neurons^21^. Moreover, the CS-inhibited neurons showed correlative firings with conditioning-potentiated neurons, especially extinction-resistant neurons, suggesting that CS-inhibited neurons contribute to the extinction of conditioned fear and the regulation of fear memory after extinction by influencing the neighboring CS-excited neurons that encode fear memory. CS-evoked inhibition may result from recruitment of two independent populations of inhibitory neurons after conditioning or extinction. One such population of inhibitory neurons, activated after extinction, may represent the parvalbumin and cholecystokinin-positive interneurons that inhibit fear neurons during extinction^32^. The enhanced activity of the other population of inhibitory neurons, activated after conditioning, may be involved in the increased synchronization of the activities between neurons in the basolateral amygdala after conditioning^33,34^.

Lesioning or inactivation of the BA_L_ alone before extinction does not affect the expression of conditioned fear, unless both the BA_L_ and the basomedial part of the basolateral amygdala are damaged^17–19^. Inactivation of the BA_L_ attenuates the expression of conditioned fear only after extinction^17–19,21^. This is consistent with the proposal that the balance between the activities of fear and extinction neurons encodes fear states after extinction. Thus, researchers have investigated whether inactivation of these neurons produces predictable changes in fear behaviors. However, it is extremely difficult to selectively inactivate fear or extinction neurons, mainly because selective markers for these neurons are not available. Instead, Senn et al. optogenetically labeled two types of BA_L_ neurons^20^: Prelimbic prefrontal cortex (PL)-projecting BA_L_ neurons and infralimbic prefrontal cortex (IL)-projecting BA_L_ neurons. The PL-projecting and IL-projecting BA_L_ neurons appear to include fear and extinction neurons, respectively; however, the majority of the PL-projecting and IL-projecting neurons have not been characterized. Although fear extinction is attenuated by optogenetic inactivation of IL-projecting BA_L_ neurons, this does not mean that extinction neurons are required for fear extinction. It is also possible that extinction-inhibited neurons, which resemble extinction neurons, also play a critical role in fear extinction, as shown in the present study. Further studies are needed on the selective inactivation of different types of neurons to determine their contributions to fear states after extinction.

In several recent studies, CS-inhibited neurons have been observed in the BA_L_ in Pavlovian conditioning^26,27,35,36^; however, these studies also included neurons in the lateral amygdala. The proportion of CS-inhibited neurons in our study (15%) is less than that reported in the previous studies (up to 40–50% after conditioning). A major difference in the experimental conditions is the type of US; a painful electrical stimulus was used in the present study, whereas quinine or air puffing was used in the previous studies. It is unclear whether different types of US cause differences in the proportion of CS-inhibited neurons after conditioning. It would be interesting to clarify the precise roles of the CS-inhibited BA_L_ neurons in encoding various emotional states by tracking the activities of CS-inhibited neurons longitudinally throughout different behavioral sessions.

An et al. demonstrated that extinction neurons acquire CS-responsiveness after a single session of extinction, and lose their responses when extinction is repeated. Intriguingly, the present study has shown that the CS-inhibited BA_L_ neurons, which acquire CS-responsiveness after a single session of extinction, lose their responses when extinction is repeated. Together, these findings support the previous proposal that the inhibition mechanism primarily operates in the early phase of extinction^22^. Additionally, conditioning-inhibited neurons which acquire CS-responsiveness after conditioning, progressively lose their CS-responsiveness when extinction is repeated (see Fig. 6). Although these neurons resemble fear neurons, there is a difference in the pattern of their activity changes during conditioning and extinction; fear neurons acquire CS responsiveness after conditioning and lose them abruptly after a single session of extinction. Currently, the relationship between fear neurons and conditioning-inhibited BA_L_ neurons is unclear. Together, our findings suggest that the inhibitory mechanisms shown in this study are also limited in the early phase of extinction.

We here present a more comprehensive view of how the activities of distinct BA_L_ neurons change during fear conditioning and extinction. In particular, we found new types of CS-inhibited neurons whose activities are dynamically modified in fear conditioning and extinction. Although the CS-excited neurons outnumber the CS-inhibited neurons, it is possible that CS-inhibited neurons play a distinct role in regulating fear states after extinction, possibly through the functional connectivity with CS-excited neurons which encode fear memory. Our findings indicate the presence of inhibitory neurons that are recruited after conditioning and extinction. In addition, many previous studies have reported sex differences in fear learning and subsequent extinction. It would be interesting to see how the portion of conditioning-potentiated neurons and extinction-potentiated neurons are different between male and female^37–39^.

## Materials and methods

### Animals

Eight-week-old male Sprague–Dawley rats were individually housed under an inverted 12-h light/dark cycle (lights off at 09:00) and were provided with food and water ad libitum. Behavioral training was conducted during the dark portion of the cycle. All procedures were approved by the Institute of Laboratory Animal Resources at Seoul National University (SNU-150114-2-1), which was advised by the Animal Ethics Committee. All experiments were performed in accordance with the guidelines and regulations made by the Animal Ethics Committee.

### Behavioral apparatus

All experiments in the present study were performed as described previously^22,23^ with some modifications. Fear conditioning and extinction took place in two different contexts (contexts A and B) to minimize the influence of contextual associations. Context A was a rectangular Plexiglass box with a metal grid floor connected to an electrical current source (Coulbourn Instruments, Allentown, PA, USA), which was placed inside a sound-attenuating chamber. The chamber was illuminated with white light and cleaned with 70% ethanol. Context B was a cylindrical Plexiglass chamber with a metal grid or a flat Formica floor and cleaned with 1% acetic acid. All the training sessions were videotaped, and the conditioned freezing was quantified by trained observers. The animals were considered frozen when there was no movement except for respiratory activity for 2 s during the 30-s CS presentation. The total freezing time was normalized to the duration of the CS presentation.

### Behavioral procedures

A total of 41 rats (8 weeks old, 290–310 g) underwent the surgical and behavioral procedures as described. The data from 27 rats were published in a previous study^22^. The rats were anesthetized with sodium pentobarbital (50 mg/kg, i.p.) and secured in a stereotaxic frame (Stoelting Co., Wood Dale, IL, USA). Anesthesia was maintained with isoflurane (1–1.5%) in O_2_, and fixed-wire electrodes were implanted into the BA_L_ 2.85 mm posterior to bregma, 5.0–5.1 mm lateral to midline, and 8.8 mm deep to the cortical surface. The electrodes consisted of eight individually insulated nichrome microwires (50 μm outer diameter, impedance 0.5–1 MΩ at 1 kHz; California Fine Wire, Grover Beach, CA, USA) contained in a 21-gauge stainless steel guide cannula. The implant was secured using dental cement (Vertex-dental, Zeist, Netherlands). An analgesic (Metacam, Boehringer Ingelheim, Germany) and an antibiotic were also applied. Single units were recorded using a Plexon MAP system (Dallas, TX, USA), as previously described^22,23^. After 6–7 days of recovery, the rats were handled for 10–20 min twice a day for 2 days. The rats were habituated to context A as follows: they were first exposed to the context for 10 min, and 8 h later, they were exposed to four CSs in that context. On day 1, the rats were exposed to five presentations of the CS to determine their basal neural responses to the CS (Pre-FC). The CS was a series of twenty-seven 7.5-kHz pure-tone pips, 200 ms in duration and repeated at 0.9 Hz, with an 85-dB sound pressure level. Fear conditioning (FC) was conducted 5 min later by pairing the CS with a mild electric foot shock (0.6 mA, 1 s, 5 CS/US pairings; inter-trial interval: 80–120 s). The first extinction training took place 8 h after conditioning in context B (EX1), in which the rats were presented with 20 non-reinforced CS presentations. Two additional extinction sessions were conducted on the following day (EX2, 3). The first 5 CSs of each extinction session were considered to measure retention of the previous training (Post-FC, Post-EX1, Post-EX2). On day 3, the behavioral and neuronal outcomes of the three extinction sessions were observed in a short test session of five CSs (Post-EX3). The rats were considered to be frozen when no movement except for respiratory activity was observed for 2 s during CS presentation. A trained experimenter manually measured freezing when the rats showed no movement while the CS was sounding. The total freezing time was normalized to the duration of the CS presentation.

### Single-unit spike sorting and analysis

Unit sorting and analysis were performed as in previous studies^22,23^. Unit discrimination was performed using Offline Sorter (OFS, Plexon, Dallas, TX, USA). All the waveforms were plotted in a principal component space, and clusters consisting of similar waveforms were first defined automatically and then verified manually. A cluster of waveforms distinct from other clusters in the principal component space and showing a clear refractory period > 1 ms was considered to be generated by a single neuron. Single unit isolation was graded using two statistical parameters, J3 and the Davies–Bouldin validity metric (DB), and neurons with low grades were discarded. J3 reflects the ratio of the between-cluster separation to the within-cluster density calculated in a principal component space, and the DB is the ratio between the sum of within-cluster density to the degree of separation between clusters. Thus, a high J3 and a low DB value indicate a compact, well-separated unit cluster^40^. The long-term stability of a single-unit isolation was first determined using Wavetracker (Plexon, Dallas, TX, USA), in which the principal component space-cylinders of a unit recorded from different sessions were plotted^21–23,41^. A straight cylinder suggests that the clusters of a unit have a similar principal-component composition, and that the same set of single units was recorded during the entire training session. Next, we calculated the values of the linear correlation (r) between the template waveforms and those obtained over the entire set of behavioral sessions^42^ to evaluate the similarity of waveform shapes. Only stable units (r > 0.92) were considered for further analysis. A total of 204 stable and high signal-to-noise units in the BA_L_ was used for the results in this study, and 130 units out of the 204 units have been published in our previous study^22^.

To investigate the effects of training on the BA_L_ neurons, the CS-evoked neural activities were normalized using a standard z-score transformation with a bin size of 20 ms. The unit responses to the first five CS, consisting of 135 tone pips, were first averaged and normalized to the baseline (firing rates during the 500 ms preceding the 135 tone pips). Z-score PETHs (peri-event time histograms) of averaged CS responses were constructed for each neuron and then averaged for every CS. The mean z-values in the interval 0–100 ms following CS-onset, i.e., from the first five CSs of each session, were compared at multiple time points throughout behavioral training. A neuron was determined to be CS-responsive if it showed significant excitatory/inhibitory responses within 100 ms following CS-onset compared to baseline (rank-sum test, *p* < 0.05). The onset latency of the CS-evoked responses was defined as the first bin to become significantly different from baseline. The basal firing rates were estimated from the Pre-CS intervals of the session. CS-responsive neurons were categorized into sub-populations based on changes in their CS-responses throughout the behavioral training. Neurons that showed significant CS-responses in Post-FC were classified as conditioning-potentiated or conditioning-inhibited neurons and they were further categorized into subgroups according to their CS-responses in the preceding and subsequent sessions. Neurons that showed significant CS-responses in Post-EX1 were classified as extinction-potentiated or extinction-inhibited neurons. Additionally, in order to determine whether false positive responses which were not related to the CS would contribute to the CS-responses of BA_L_ neurons, we randomly sampled neuronal firings of all the recorded neurons before CS presentation and examined firing changes in response to hypothetical CSs across the entire behavioral sessions using the same analyses that were used to detect CS responsiveness of BA_L_ neurons (Supplementary Figure S6). We rarely found firing pattern changes similar to the results shown herein, indicating that false positive responses did not contribute to the results.

Cross-correlation analysis was used to examine function connectivity between CS-responsive neurons. Neuronal pairs were obtained and correlated firings were examined only if more than two CS-responsive neurons were observed in a rat. Cross-correlograms were drawn from neuronal spikes of target neurons in accordance with spikes of reference neurons using NeuroExplorer (Nex Technologies, Colorado Springs, CO, USA). If any bin (bin size, 0.5 ms) within 4 ms in the cross-correlogram of a neuronal pair was greater than the confidence line (99%), which was calculated based on the confidence intervals for the expected of a Poisson random variable, the pair was considered to show significant correlative firings^29–31^.

### Histology

At the end of the experiments, the rats were anesthetized with urethane (1 g/kg, i.p.) and electrolytic lesions were made by passing a current (10 μA, 5–20 s) through the recording microwires. This procedure enabled the locations of their recording sites to be determined. The animals were then transcardially perfused with 0.9% saline solution and 10% buffered formalin. The brains were removed and post-fixed overnight. Coronal sections (100 μm thick) were obtained using a vibroslicer (NVSL; World Precision Instruments, Sarasota, FL, USA) and stained with cresyl violet. The placement of the recording microwires was examined under light microscopy and compared to rat brain atlases^43,44^. The figure was constructed using an open rat brain atlas, Brain maps (Fig. 1g)^44^.

### Statistical analysis

Statistical significance was tested using the Mann–Whitney *U* test for comparing two groups and the Friedman test for comparing three or more groups. For post-hoc multiple comparisons, Dunn’s test with an adjusted false discovery rate was used. Pearson’s correlation test was used to determine the correlations between freezing behavior and neuronal activity. The data for all the training and post-training experiments included samples from three or more rats. Error bars represent the standard error of the mean. A *p* value < 0.05 was considered statistically significant. The present study was carried out in compliance with the ARRIVE guidelines (http://www.nc3rs.org.uk/page.asp?id=1357).

## Supplementary Information

Supplementary Information.

## Data Availability

The data that support the findings of this study are available from the corresponding author upon reasonable request.
