# Supplementary material for: Longitudinal recordings of single units in the basal amygdala during fear conditioning and extinction
Source: Sci Rep. 2021 May 27;11:11177. doi: 10.1038/s41598-021-90530-x (PMC8159982; doi:10.1038/s41598-021-90530-x)
Supplement: Supplementary file 1 — Supplementary Information. [file 41598_2021_90530_MOESM1_ESM.pdf]

Longitudinal recordings of single units in the basal amygdala during fear conditioning and extinction

Junghwa Lee[1], Bobae An[2] *, and Sukwoo Choi[1]

[1] School of Biological Sciences, College of Natural Sciences, Seoul National University, Seoul, Korea (ROK)

[2] McGovern Institute for Brain Research, Massachusetts Institute of Technology, USA

* **Correspondence:** Bobae An (bobaean@gmail.com) or Sukwoo Choi (lead contact: sukwoo12@snu.ac.kr)

Supplementary Figure S1

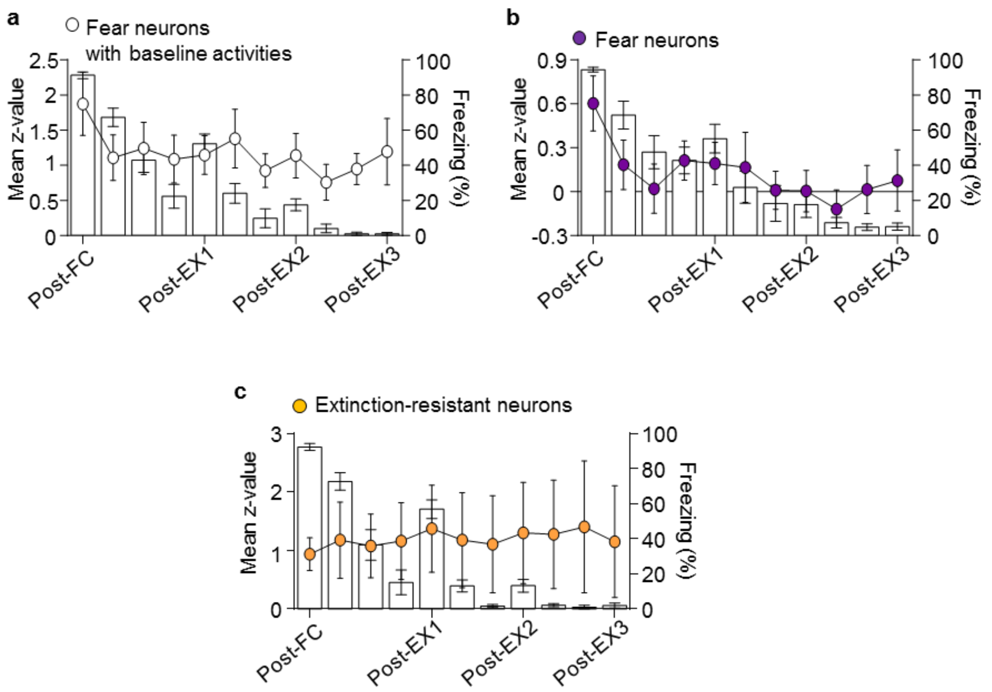

Supplementary Figure S2

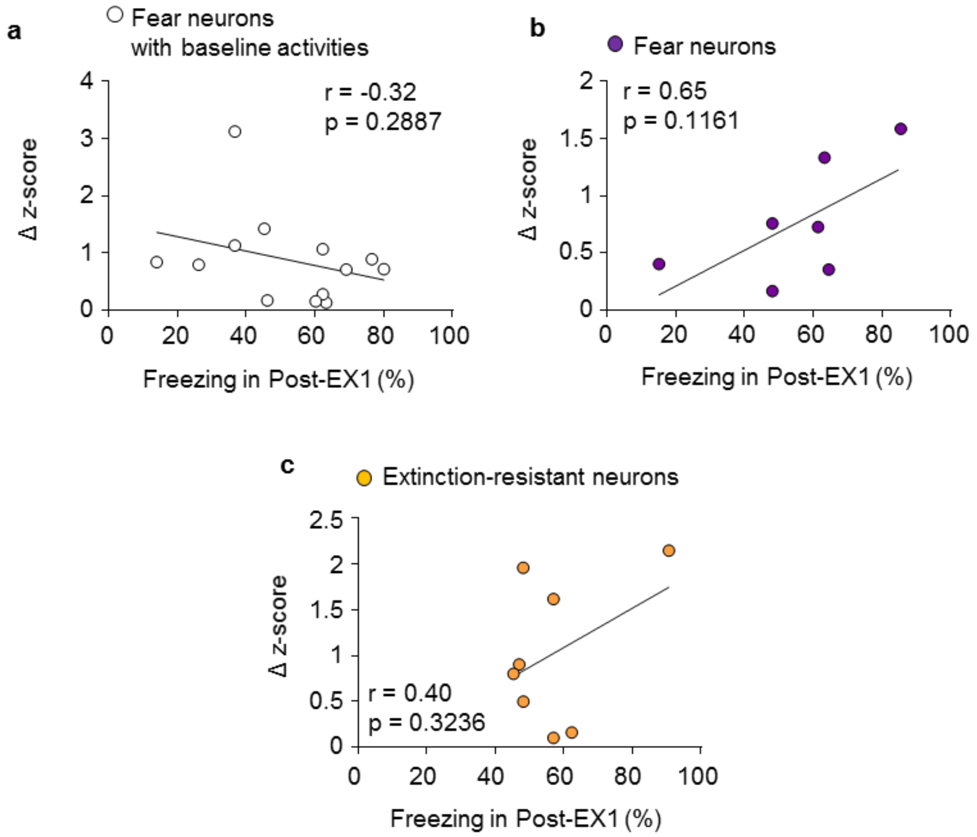

Supplementary Figure S3

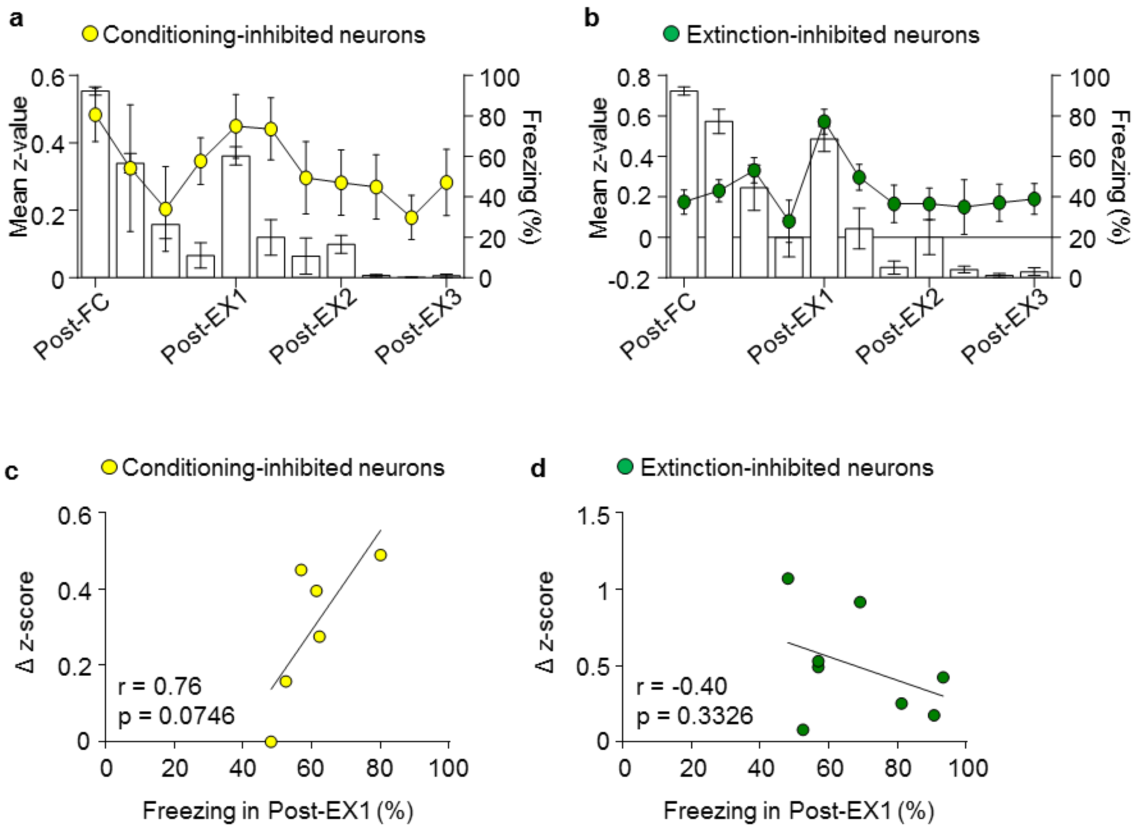

Supplementary Figure S4

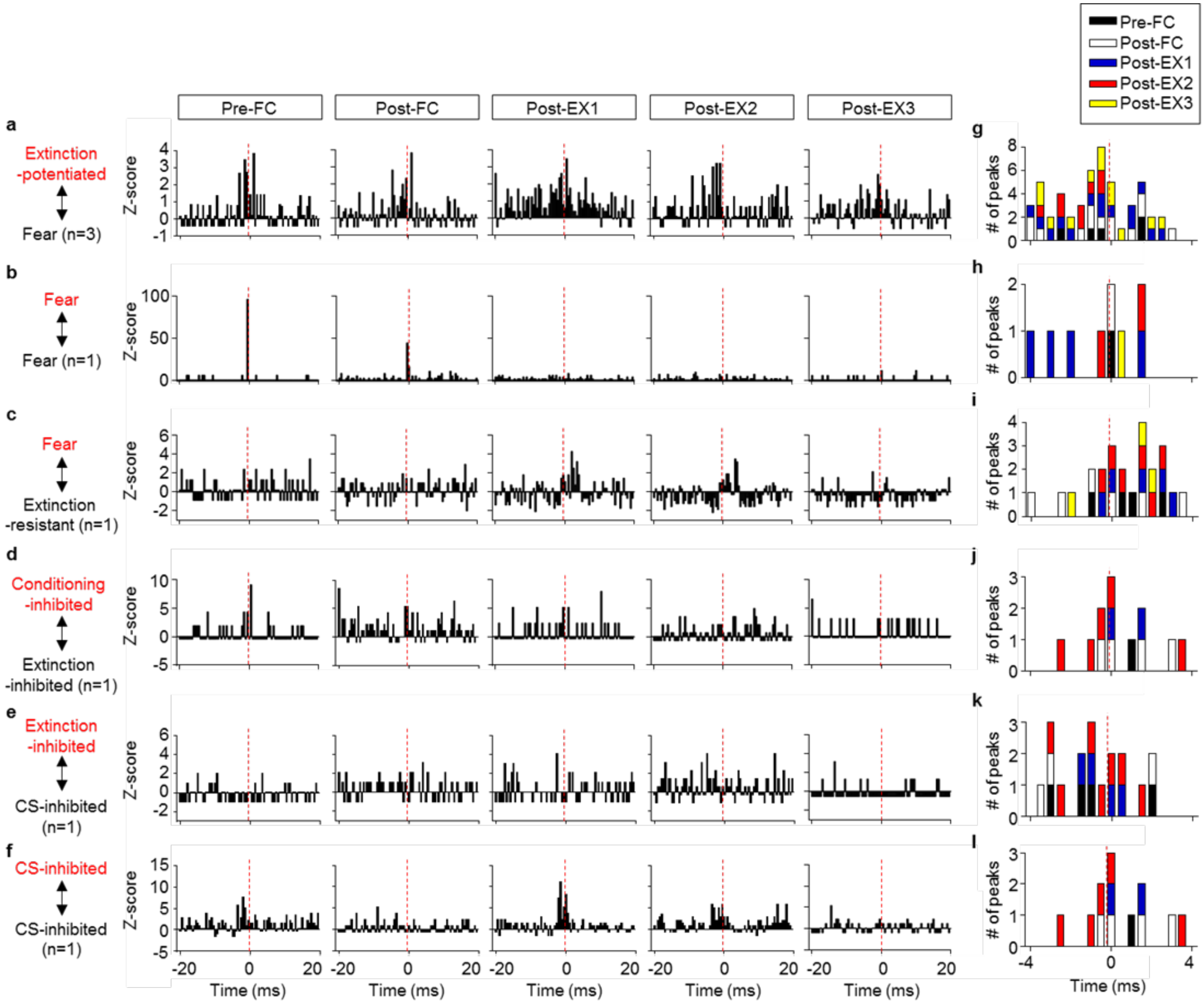

Supplementary Figure S5

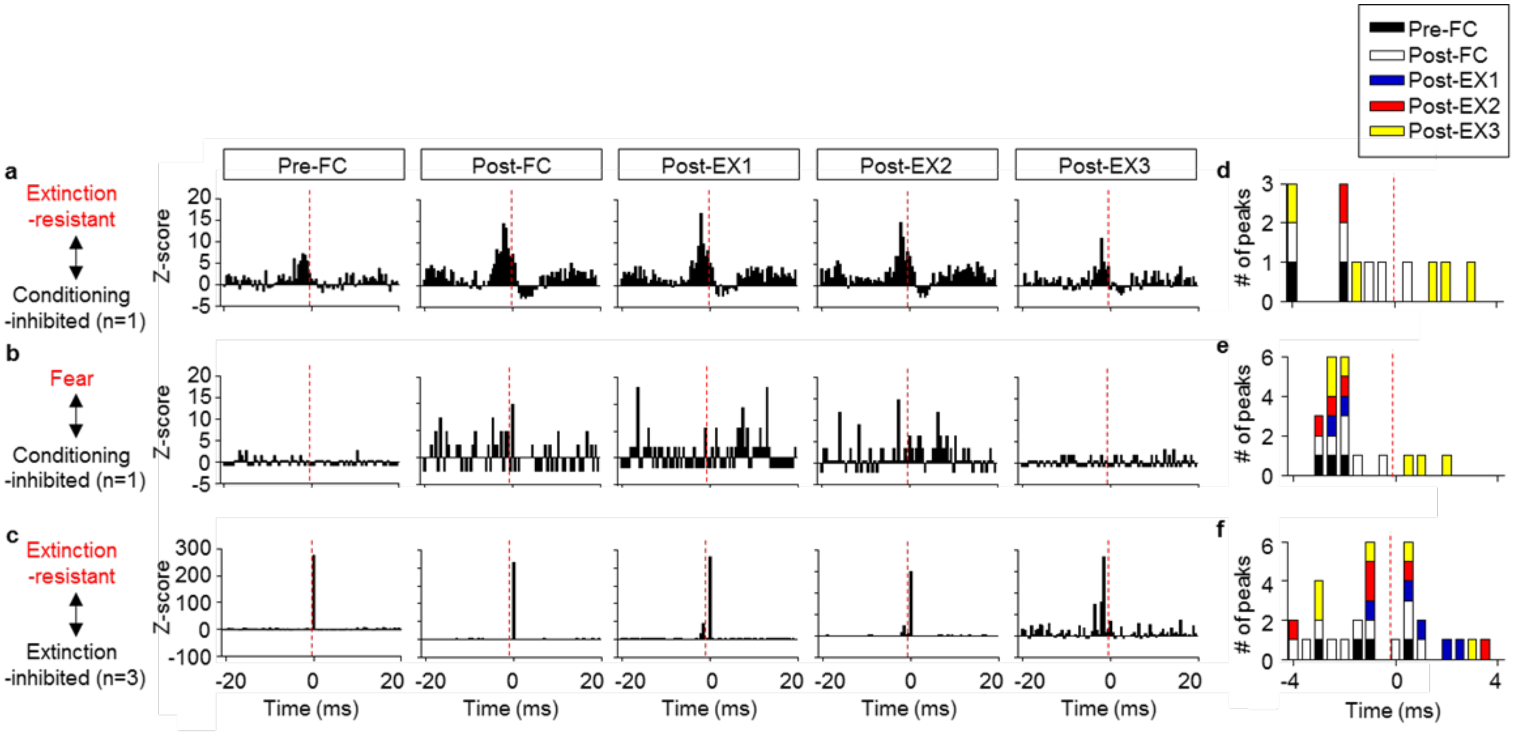

Supplementary Figure S6

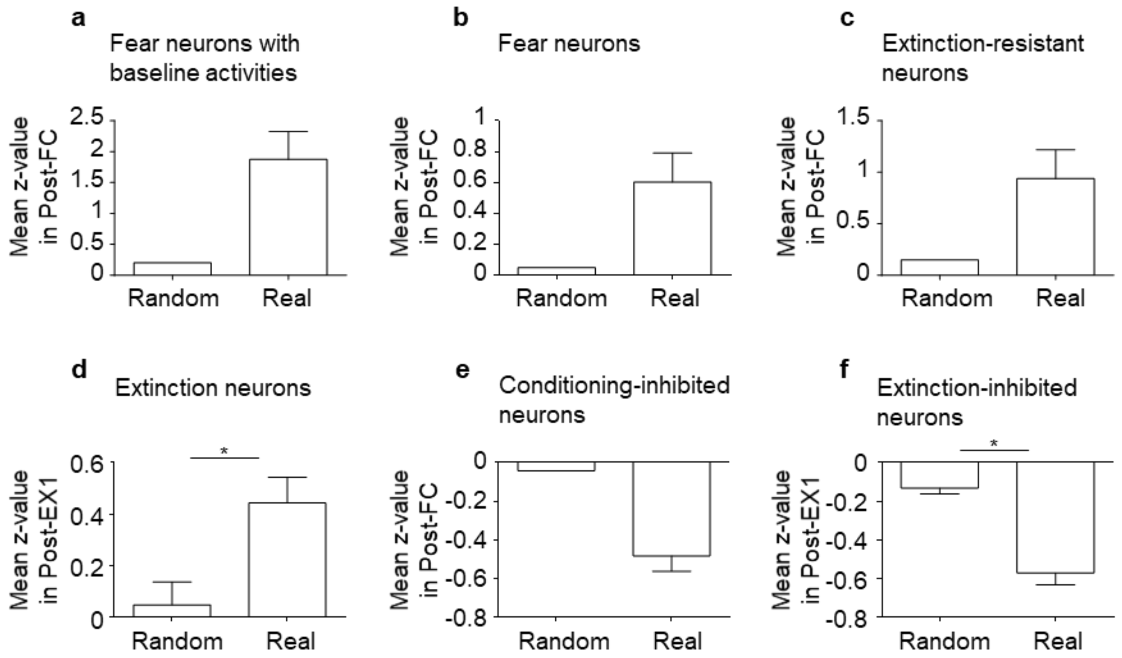

Supplementary Figure S7

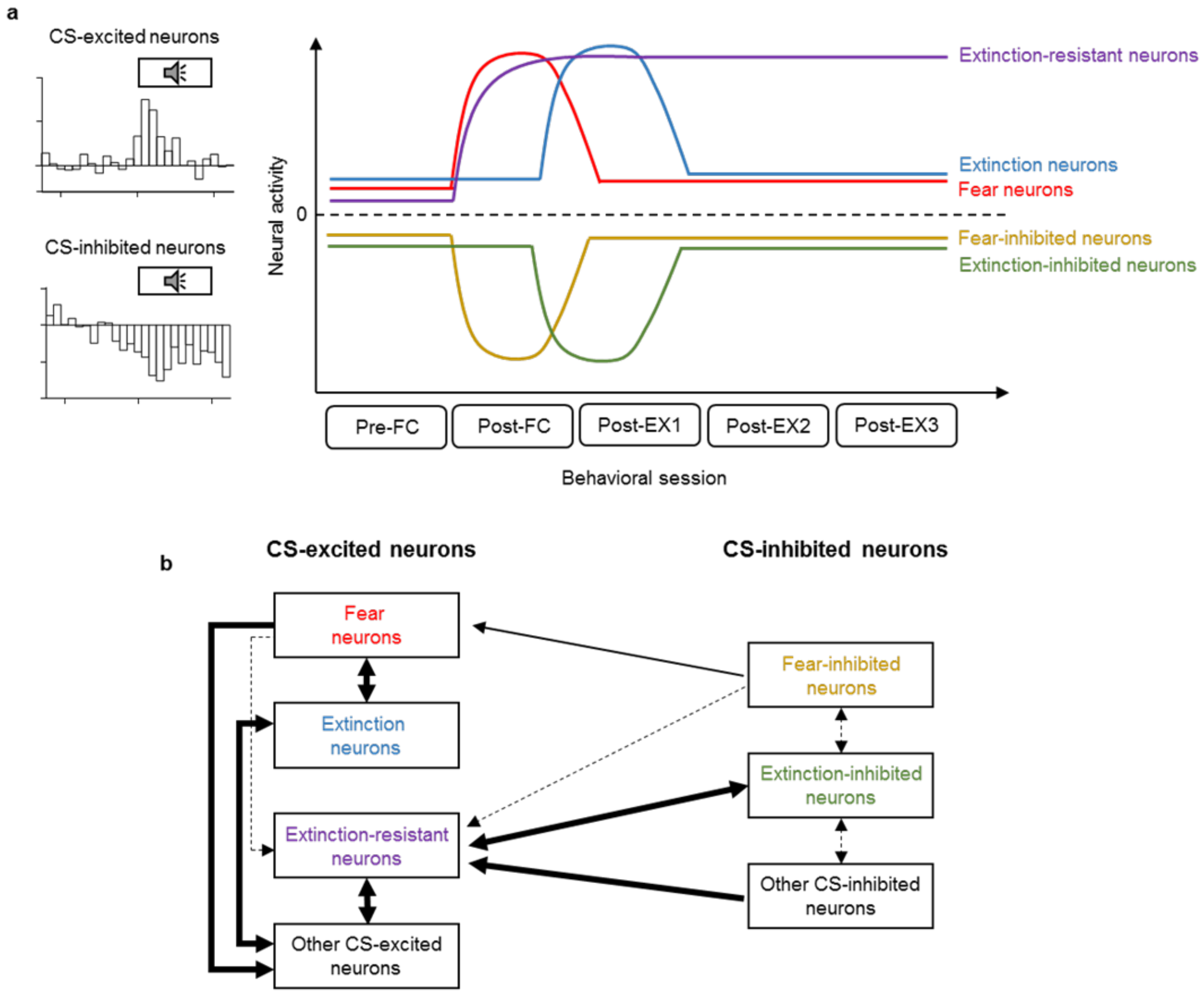

Supplementary information

Supplementary Figure S1. The averaged time courses of CS-evoked activity in fear neurons with baseline activities and fear neurons are similar to those of freezing during extinction, however, extinction-resistant neurons show persistent CS-evoked activity irrespective of the amount of extinction. (**a-c**) Averaged time courses of the freezing responses and z-scored neuronal activity of (**a**) fear neurons with baseline activities, (**b**) fear neurons, and (**c**) extinction-resistant neurons. Each bar represents averaged freezing response during five consecutive CS presentations, and each point represents averaged z-scores during five consecutive CS presentations.

Supplementary Figure S2. Fear neurons and extinction-resistant neurons in Post-FC exhibit proportional correlations with fear response after the first extinction session (Post-EX1), whereas fear neurons with baseline activities in Post-FC show an inverse correlation with the fear response after the first extinction session (Post-EX1). (**a-c**) Correlation analysis between the neural responses in Post-FC and freezing behavior in Post-EX1 in each group ($r = -0.32$, $p = 0.2887$ for **a**; $r = 0.65$, $p = 0.1161$ for **b**; $r = 0.40$, $p = 0.3236$ for **c**, Pearson's correlation).

Supplementary Figure S3. The activities of conditioning-inhibited and extinction-inhibited neurons correlate with the freezing response. (**a**) Averaged time courses of freezing responses and neuronal activity (z-scores) of conditioning-inhibited neurons. (**b**) Averaged time courses of freezing responses and neuronal activity (z-scores) of extinction-inhibited neurons. In (**a**) and (**b**), each bar represents averaged freezing responses in five consecutive CS presentations, and

each point represents averaged z-scores during five consecutive CS presentations. (**c, d**) Correlation analysis between the neural responses and freezing behavior. Freezing behavior in Post-EX1 was positively correlated with activities of conditioning-inhibited neurons ($r = 0.76$, $p = 0.0746$ for **c**, Pearson's correlation) and was negatively correlated with extinction-inhibited neurons ($r = -0.40$, $p = 0.3326$ for **d**, Pearson's correlation), but both correlations were not significant.

Supplementary Figure S4. Functional connectivity between CS-excited neurons or CS-inhibited neurons. Z-score cross-correlogram and the distribution of the time of significant peaks in the cross-correlogram between (**a, g**) extinction-potentiated neurons and fear neurons ($n = 3$, extinction-potentiated neurons as references and fear neurons as targets), (**b, h**) fear neurons ($n = 1$, fear neuron as a target and the other as a reference), (**c, i**) a fear neuron and an extinction-resistant neuron ($n = 1$, fear neuron as a reference), (**d, j**) a conditioning-inhibited neuron and an extinction-inhibited neuron ($n = 1$, conditioning-inhibited neuron as a reference), (**e, k**) an extinction-inhibited neuron and a CS-inhibited neuron ($n = 1$, extinction-inhibited neuron as a reference), (**f, l**) CS-inhibited neurons ($n = 1$, CS-inhibited neuron as a target). Connectivity was defined as significant if there is any peak > 99% confidence index within 4 ms in the cross-correlogram and the significant peaks were counted. Positive peaks in the cross-correlogram mean firings of the reference neuron preceded the target neuron.

Supplementary Figure S5. Functional connectivity between CS-excited neurons and CS-inhibited neurons. Z-score cross-correlogram and the distribution of the time of significant peaks in the cross-correlogram between (**a, d**) an extinction-resistant neuron and a conditioning-inhibited neuron (n = 1, extinction-resistant neuron as a reference and the other as a target), (**b, e**) fear neurons and conditioning-inhibited neurons (n = 2, fear neurons as references and the conditioning-inhibited neurons as targets), (**c, f**) extinction-resistant neurons and extinction-inhibited neurons (n = 3, extinction-resistant neurons as references) throughout fear conditioning and extinction.

Supplementary Figure S6. Comparison between real CS-responses of BA_L neuronal sub-populations and non-CS-related randomly sampled firings. To determine whether false positive responses which were not related to the CS would contribute to CS-responses of BA_L neurons, we randomly sampled neuronal firings of all the recorded neurons before CS presentation and examined firing changes in response to hypothetical CSs across the entire behavioral sessions using the same analyses that were used to detect CS responsiveness of BA_L neurons. Any random firing that changed the firings patterns across behavioral training similarly to the distinct BA_L neuronal sub-populations was compared with CS-responses of the particular sub-populations. (**a**) One random firing showed similar changes in firings across behavioral sessions to fear neurons with baseline activities; the random firing showed significant responses in Pre-FC and Post-FC. However, the mean firings in Post-FC were much smaller than CS-responses of fear neurons with baseline activities. Similar comparisons were conducted between (**b**) Fear neurons and one random firing, (**c**) Extinction-resistant neurons and one random firing, (**d**) Extinction neurons and three random firings ($p = 0.0412$, Mann–Whitney test), (**e**) Conditioning-

inhibited neurons and one random firing, (**f**) Extinction-inhibited neurons and two random firings ($p = 0.0444$, Mann–Whitney test).

Supplementary Figure S7. (**a**) A schematic of distinct BA_L neurons in fear conditioning and extinction. Changes in neural activities of distinct BA_L neurons that showed excitation or inhibition to the CS are depicted throughout the behavioral training. Fear neurons and conditioning-inhibited neurons displayed CS-responses after fear conditioning and lost the responses following extinction. Extinction-resistant neurons showed increased CS-responses after fear conditioning and retained CS-responses even after multiple extinction. Extinction neurons and extinction-inhibited neurons displayed CS-responses after the first extinction and lost CS-responses after multiple extinction. (**b**) A schematic of various types of functional connectivity between CS-responsive neurons within the BA_L. Various types of significant functional connectivity are depicted as lines between distinct BA_L neurons, and the number of neuronal pairs is depicted as the thickness of the lines. Activities of the CS-excited neurons showed various types of correlative firings with other CS-excited neurons. CS-inhibited neurons displayed correlative firings mostly with conditioning-potentiated neurons, especially extinction-resistant neurons.
